# First isolation and whole-genome sequencing of a *Shewanella algae* strain from a swine farm in Brazil

**DOI:** 10.1186/s12866-020-02040-x

**Published:** 2020-11-24

**Authors:** Vinicius Buiatte de Andrade Alves, Eneas Carvalho, Paloma Alonso Madureira, Elizangela Domenis Marino, Andreia Cristina Nakashima Vaz, Ana Maria Centola Vidal, Vera Letticie de Azevedo Ruiz

**Affiliations:** 1 Universidade de Sao Paulo, Faculty of Animal Science and Food Engineering, Laboratorio de Doenças Infectoparasitarias de Animais Domesticos, 225 Duque de Caxias Av., Jardim Elite, Pirassununga, SP 13635-900 Brazil; 2 Instituto Butantan, Laboratory of Bacteriology, 1500 Vital Brasil Av., Butantan, Sao Paulo, SP 05503-900 Brazil

**Keywords:** Genome, *Shewanella*, Taxonomy, Swine, Resistance

## Abstract

**Background:**

Infections caused by *Shewanella* spp. have been increasingly reported worldwide. The advances in genomic sciences have enabled better understanding about the taxonomy and epidemiology of this agent. However, the scarcity of DNA sequencing data is still an obstacle for understanding the genus and its association with infections in humans and animals.

**Results:**

In this study, we report the first isolation and whole-genome sequencing of a *Shewanella algae* strain from a swine farm in Brazil using the boot sock method, as well as the resistance profile of this strain to antimicrobials. The isolate was first identified as *Shewanella putrefaciens*, but after whole-genome sequencing it showed greater similarity with *Shewanella algae*. The strain showed resistance to 46.7% of the antimicrobials tested, and 26 resistance genes were identified in the genome.

**Conclusions:**

This report supports research made with *Shewanella* spp. and gives a step forward for understanding its taxonomy and epidemiology. It also highlights the risk of emerging pathogens with high resistance to antimicrobial formulas that are important to public health.

**Supplementary Information:**

The online version contains supplementary material available at 10.1186/s12866-020-02040-x.

## Background

*Shewanella* spp. are Gram-negative bacteria mostly found in marine environments. Although infections can be considered rare, the number of reports have increased in recent years [1–7].. The species reported most frequently in the literature are *Shewanella algae* and *Shewanella putrefaciens* [8].

These bacteria were first isolated as part of the genus *Achromobacter* in 1931 and characterized as *Achromobacter putrefaciens* [9]*.* Later, due to advances in molecular biology, *Shewanella* was assigned as a new genus in the family Vibrionaceae [10]. More recently it is recognized as part of the family *Shewanellaceae* [11]. Due to its great genomic diversity, some strains can be classified under this group based on genetic data, even though they are phenotypically different [8].

The genus was recently revised by Thorell et al. (2019) [12] based on the genome sequences of strains described in previous studies. More than 48% of the strains (64 out of 131) were reassigned to different species or sub-species after the analysis. This reinforces the importance of research involving isolation and genomic characterization to have a better understanding of the bacteria and their epidemiology.

*Shewanella* spp. are believed to be susceptible to most antibiotics used in human medicine, such as third-generation cephalosporins, gentamicin and ciprofloxacin [7]. However, recent studies have shown an increasing resistance of new isolates [1, 13, 14]. This resistance, in addition to the increasing number of infections, underscores the importance of research focusing on these bacteria and how to prevent new infections.

In this study, we report the first isolation of a *Shewanella algae* strain from a swine farm in Brazil using the boot sock method. The isolation happened unexpectedly through a protocol designed for *Salmonella* spp.. We also report the whole-genome sequence and describe the antimicrobial susceptibility profile in order to improve the understanding of the genus.

## Results and discussion

In this study, a *Shewanella algae* strain was isolated from a pen’s floor of a swine farm in Brazil. To the best of our knowledge, this is the first report of isolation in a swine production facility.

Infectious caused by *Shewanella* spp. have been increasingly reported in humans. Since this microorganism is widely found in marine environments, many human cases have been linked to seawater exposure prior to the infection. *Shewanella* spp. are considered opportunistic bacteria, therefore, infections frequently occur in individuals who are immunocompromised or with pre-existing conditions [15]. Although the origin of the isolated strain remains unknown, the presence of this resistant zoonotic microorganism at such a different environment may represent a threat to the employees that may be exposed to it daily.

### Shewanella algae may grow in culture media for Enterobacteriaceae and can be misidentified as Shewanella putrefaciens

The strain was isolated after incubation in Tetrathionate broth and recovered from Hektoen agar. The selected colonies were submitted to Triple Sugar Iron and Lysine Iron Agar tests and became suggestive for *Salmonella* spp.. However, this hypothesis was discarded since they were oxidase-positive. Biochemical identification of the isolate was carried out using the API 20E kit (bioMérieux®, France). As a result, the strain was identified as *Shewanella putrefaciens* group, with an excellent identification (99.9%), which was later characterized as *Shewanella algae* by whole-genome sequencing.

*Shewanella algae* have some biochemical traits that differ from *Shewanella putrefaciens*, such as nitrite reduction, the ability to grow in higher temperatures and under higher concentrations of sodium chloride (NaCl). The colonies have a mucoid aspect, and they are hemolytic in blood agar. However, strains can be misidentified as *Shewanella putrefaciens* because many commercial identification systems, such as the API 20E (bioMérieux®, France) and API 20NE (bioMérieux®, France), do not contain *S. algae* in their database [16]. Botelho-Nevers et al. (2005) [17] also misidentified a *S. algae* strain as *S. putrefaciens* after biochemical identification. These findings highlight the importance of further investigation towards correct identification of the strains.

In addition, the results show that *Shewanella algae* has the ability to grow in culture media designed for the isolation of *Enterobacteriaceae*, and the morphological aspects of its colonies may incorrectly lead to classifying this bacteria as *Salmonella* spp. [18]. Therefore, these findings reinforce the necessity of using biochemical, serological, and molecular testing for differentiating *Salmonella* spp. from other bacteria.

### Whole-genome sequencing reveals greater similarity of the strain with *Shewanella algae*

**Fig. 1:**
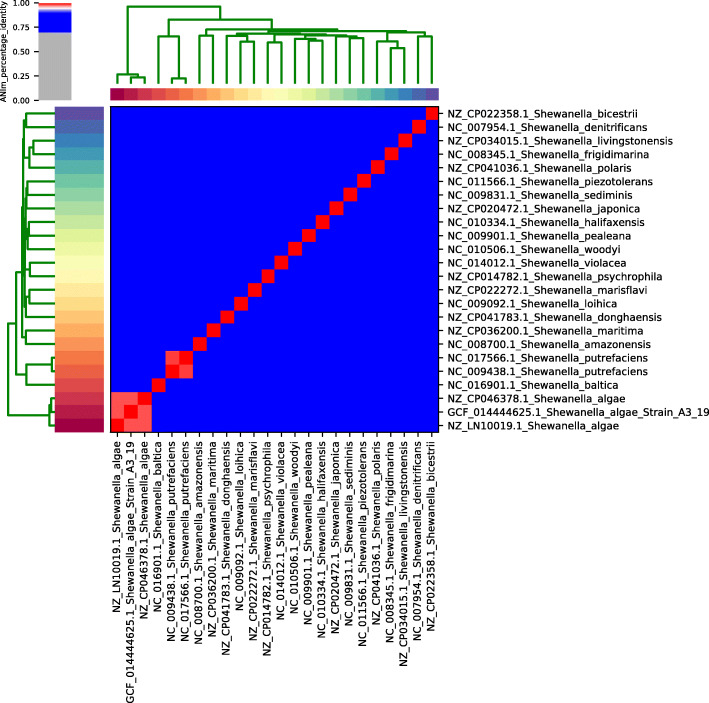
Average Nucleotide Identity (ANI) of the isolated strain to 21 *Shewanella* species. A heatmap and a hierarchical clustering are shown. Pairs of genomes with ANI > 95% can be considered as from the same species. Legend: NGS_9_1_Montagem2: *S. algae*: SAMN15541815; *S. algae*: NZ_CP046378.1; *S. algae*_2: NZ_LN10019.1; *S. amazonensis*: NC_008700.1; *S. baltica*: NC_016901.1; *S. bicestrii*: NZ_CP022358.1; *S. denitrificans*: NC_007954.1; *S. donghaensis*: NZ_CP041783.1; S*. frigidimarina*: NC_008345.1; *S. halifaxensis*: NC_010334.1; *S. japonica*: NZ_CP020472.1; *S. livingstonensis*: NZ_ CP034015.1; *S. loihica*: NC_009092.1; *S. marisflavi*: NZ_CP022272.1; *S. maritima*: NZ_CP036200.1; *S. pealeana*: NC_009901.1; *S. piezotolerans*: NC_011566.1; *S. polaris*: NZ_CP041036.1; *S. psychrophila*: NZ_CP014782.1; *S. putrefaciens*: NC_009438.1; *S. putrefaciens*_2: NC_017566.1; *S. sediminis*: NC_009831.1; *S. violacea*: NC_014012.1; *S. woodyi*: NC_010506.1

**Fig. 2:**
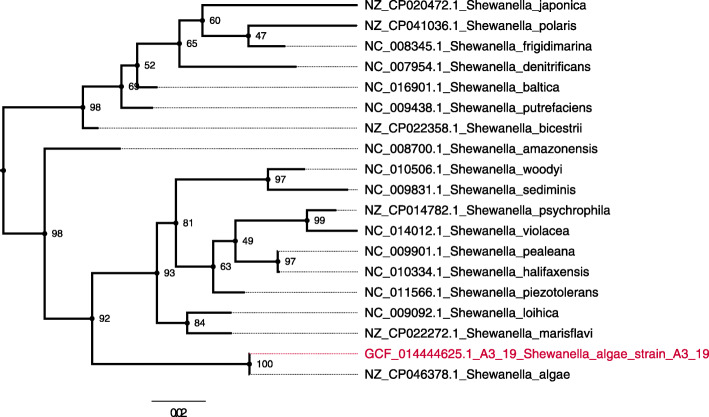
Phylogenetic tree based on 16S-rDNA. The isolate of *Shewanella algae* A3/19 is highlighted in red color

Following identification, the strain was subjected to whole-genome sequencing, and characterized as *Shewanella algae*. The draft-genome (276 contigs > 200 bases, N50 = 88.103 bases) has 4,944,286 bases, in which 4265 genes were detected with a GC-content (guanine-cytosine content) of 53.15%. The draft-genome of the isolate, named A3/19, was deposited on GenBank under the accession number GCA_014444625.1. When the isolate’s draft-genome was compared to 21 other species of *Shewanella* retrieved from the GenBank database, it showed an ANI > 98% to two species of *Shewanella algae* against ANI < 84% of similarity to two *Shewanella putrefaciens* genomes (Fig. 1). Since ANI > 95% is a robust indicator of two samples belonging to the same species [19] it can be concluded that this strain is a *Shewanella algae* species. A phylogenetic analysis based on 16S ribosomal gene sequence also indicated that the isolate A3/19 is, indeed, *Shewanella algae* (Fig. 2).

### Antimicrobial susceptibility test and detection of resistance genes

The isolated strain was resistant to 46.7% of the antibiotics tested (7 out of the 15 – POLISENSIDISC, DME®, Brazil) as shown in Table 1. *Shewanella* spp. are usually susceptible to Tetracycline, Chloramphenicol, Ciprofloxacin and Amoxicillin [6, 7, 20]. However, our strain was resistant to all of these formulas. In swine production, these antimicrobials have been broadly used as in-feed or therapeutically [21], which can possibly explain the resistance found in this study.

**Table 1:** Susceptibility of the isolate to a set of 15 antimicrobials (POLISENSIDISC 15 - DME®, Brazil)

Antimicrobial	Susceptibility
Amikacin	Susceptible
Amoxicillin/Clavulanic Acid	**Resistant**
Ampicillin	**Resistant**
Aztreonam	Susceptible
Cefazolin	**Resistant**
Cefepime	Susceptible
Cefoxitin	Susceptible
Ceftadizime	Susceptible
Ceftriaxone	Susceptible
Ciprofloxacin	**Resistant**
Chloramphenicol	**Resistant**
Gentamicin	Susceptible
Meropenem	Susceptible
Trimethroprim/Sulfamethoxazole	**Resistant**
Tetracycline	**Resistant**

In the genetic characterization, we found 26 genes similar to antimicrobial resistance genes (See Additional file 1), which predicts that this strain could be resistant to 21 antibiotics: Amoxicillin, Ampicillin, Streptomycin, Trimethoprim, Amikacin, Sulfamethoxazole, Chloramphenicol, Ciprofloxacin, Doxycycline, Cephalothin, Piperacillin, Ticarcillin, Tobramycin, Gentamicin, Ampicillin + Clavulanic acid, Amoxicillin + Clavulanic acid, Tigecycline, Imipenem, Florfenicol, Tetracycline and Minocycline. These results, based on antimicrobial susceptibility tests and whole genome sequencing, suggest that this is possibly a multidrug resistant strain.

In addition, a total of eleven genes found in the draft-genome (see Additional file 2) were similar to virulence factors, which suggests that, even though the strain was isolated from an environmental sample, it is a potentially hazardous microorganism that could cause infections in both animals and humans.

## Conclusion

This study reports the first isolation and whole-genome sequencing of a *Shewanella algae* strain from a swine production farm in Brazil. The genetic characterization was important to classify the isolate correctly, to confirm that it is a multidrug-resistant strain, and to alert for the potential of being an infective microorganism. The resistance profile found in this study highlights the risk of the indiscriminate usage of antibiotics in animal production. These findings draw attention to the power of genomic approaches for understanding *Shewanella* spp. strains in order to improve research made with these bacteria.

## Methods

The isolation of *Shewanella algae* happened unexpectedly, since their colonies can be phenotypically similar to *Salmonella* spp. in many culture media for *Enterobacteriaceae* [18]. The aim of this methodology was to correctly identify the strain through whole-genome sequencing and evaluate resistance through antimicrobial sensitivity test.

### Sample collection

The samples were collected from a farm in the State of Sao Paulo, Brazil. The pens of weaned pigs were sampled using the boot sock method on the surface of the pen floor. The boot socks were prepared in the laboratory using disposable shoe covers that were sterilized at 121 °C for 15 min. At the farm, two layers of disposable shoe covers were worn by one of our collaborators and dragged onto the surface area of the pen to reach as much area as possible. One layer was used to cover the shoes and decrease contamination, and the other to perform the collection and testing. The samples were kept in a sterile bag, and stored on ice until processing.

### Isolation and identification of strains

**Fig. 3:**
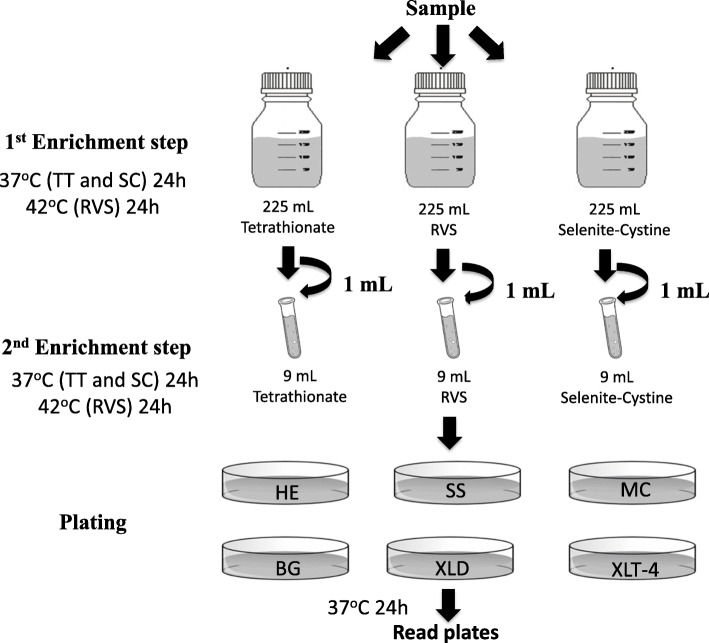
Protocol used for the isolation of *Shewanella algae* in this study

The samples were subjected to three different protocols for the isolation of *Salmonella enterica subsp. enterica*, based on the use of different culture media and growth conditions. A schematic representation of the methodology used for the isolation of the colonies is described in Fig. 3.

The enrichment step was performed twice with three different broths: Tetrathionate (TT) (Neogen®, United States), Rappaport Vassiliadis Soya (RVS) (KASVI®, Brazil) and Selenite Cystine (SC) (KASVI®, Brazil). First, samples were inoculated in glass bottles containing 225 mL of each broth and incubated at the recommended temperatures for 18–24 h. After incubation, a 1 mL aliquot was inoculated into 9 mL of broth and again incubated for 18–24 h. The samples were plated onto six different media: Hektoen (HE) (KASVI®, Brazil), Salmonella-Shigella (SS) (KASVI®, Brazil), MacConkey (MC) (KASVI®, Brazil), Brilliant-Green (BG) (KASVI®, Brazil), Xylose Lysine Deoxycholate (XLD) (KASVI®, Brazil) and Xylose Lysine Tergitol-4 (XLT-4) (KASVI®, Brazil). All media were prepared following the manufacturer’s recommendations and incubated at the suggested temperatures.

The broths used in this protocol were supplemented with a solution of Novobiocin (INLAB®, Brazil) (40 μg/mL) as suggested by Pessôa and Peixoto (1971) [22] and ISO 6579 (NBR, 2014) [23] in order to decrease competition with *Proteus* spp..

The suspected colonies were subjected to testing on Lysine Iron Agar (KASVI®, Brazil) and Triple Sugar Iron Agar (KASVI®, Brazil). This procedure is used for differentiating bacteria of the family *Enterobacteriaceae*.

The final identification was performed with API 20E (bioMérieux®, France) kit, following all manufacturer’s instructions. This biochemical system allows the identification of *Enterobacteriaceae* and other non-fastidious Gram-negative bacteria.

### Antibiogram

The sensitivity to antibiotics was tested using the POLISENSIDISC 15 (DME®, Brazil), following the product’s instructions. This system tests 15 antibiotics: Amikacin, Amoxicillin/Clavulanic Acid, Ampicillin, Aztreonam, Cefazolin, Cefepime, Cefoxitin, Ceftadizime, Ceftriaxone, Ciprofloxacin, Chloramphenicol, Gentamicin, Meropenem, Trimethroprim/Sulfamethoxazole, Tetracycline.

### Whole-genome sequencing and phylogenetic assessment

The sequencing was carried out in an Illumina Miseq platform, using kits and protocols from the manufacturer. Raw data was processed by Fastp [24]. Then, it was input to SPAdes [25] in order to assemble the bacterial genome. Annotation was carried out with NCBI Prokaryotic Genome Annotation Pipeline (PGAP), and the detection of genes related to virulence and antimicrobial resistance was performed with Abricate (Seemann T, Abricate, Github) [26], using the databases “The virulence factor database” - VFDB and ResFinder [23]. The ANI was calculated using PyANI [27].

The sequences of 16S Ribosomal genes were obtained either from Genbank or from our whole genome sequencing data. The alignment was performed with Muscle [28], and the Maximum Likelihood phylogeny was carried out using IQTree [29], selecting the best substitution model with ModelFinder [30] and testing for best branch supports with the ultrafast bootstrap [31]. The resulting tree was visualized using FigTree [32].

## Supplementary Information

**Additional file 1.** Antimicrobial resistance genes of the isolate A3/19. The list of 26 resistance genes are provided in this file with all the important information related to them.

**Additional file 2.** Virulence factors genes of the isolate A3/19. The list of 11 virulence genes are provided in this file with all the important information related to them.

## Data Availability

The dataset supporting the conclusions of this article is available in the GenBank, [GCA_014444625.1 https://www.ncbi.nlm.nih.gov/assembly/GCA_014444625.1].

## Abbreviations

GC-content: Guanine-Cytosine content
TT: Tetrathionate
RVS: Rappaport Vassiliadis Soya
SC: Selenite Cystine
HE: Hektoen
SS: Salmonella-Shigella
MC: MacConkey
BG: Brilliant-Green (BG)
XLD: Xylose Lysine Deoxycholate
XLT-4: Xylose Lysine Tergitol-4
*S. algae*: *Shewanella algae*
*S. putrefaciens*: *Shewanella putrefaciens*
*S. amazonensis*: *Shewanella amazonensis*
*S. baltica*: *Shewanella baltica*
*S. bicestrii*: *Shewanella bicestrii*
*S. denitrificans*: *Shewanella denitrificans*
*S. donghaensis*: *Shewanella donghaensis*
S*. frigidimarina*: *Shewanella frigidimarina*
*S. halifaxensis*: *Shewanella halifaxensis*
*S. japonica*: *Shewanella japonica*
*S. livingstonensis*: *Shewanella livingstonensis*
*S. loihica*: *Shewanella loihica*
*S. marisflavi*: *Shewanella marisflavi*
*S. maritima*: *Shewanella maritima*
*S. pealeana*: *Shewanella pealeana*
*S. piezotolerans*: *Shewanella piezotolerans*
*S. polaris*: *Shewanella polaris*
*S. psychrophila*: *Shewanella psychrophila*
*S. sediminis*: *Shewanella sediminis*
*S. violacea*: *Shewanella violacea*
*S. woodyi*: *Shewanella woodyi*
rDNA: ribossomal DNA
